# The Role of the Kynurenine Signaling Pathway in Different Chronic Pain Conditions and Potential Use of Therapeutic Agents

**DOI:** 10.3390/ijms21176045

**Published:** 2020-08-22

**Authors:** Filip Jovanovic, Kenneth D. Candido, Nebojsa Nick Knezevic

**Affiliations:** 1 Department of Anesthesiology, Advocate Illinois Masonic Medical Center, 836 W. Wellington Ave. Suite 4815, Chicago, IL 60657, USA; drfilipjovanovic91@gmail.com (F.J.); kdcandido1@gmail.com (K.D.C.); 2 Department of Anesthesiology, University of Illinois, Chicago, IL 60612, USA; 3 Department of Surgery, University of Illinois, Chicago, IL 60612, USA

**Keywords:** tryptophan, kynurenine, IDO-1, IDO-2, KMO, chronic pain, depression, metabolic pathway

## Abstract

Tryptophan (TRP) is an essential, aromatic amino acid catabolized by indoleamine 2,3-dioxygenase (IDO) and tryptophan 2,3-dioxygenase (TDO) enzymes into kynurenine. The IDO enzyme is expressed in peripheral tissues and the central nervous system. Another enzyme of interest in the kynurenine signaling pathway is kynurenine 3-monooxygenase (KMO). The purpose of this review is to discuss the role of TRP and the kynurenine signaling pathway in different chronic pain patients. The IDO-1, IDO-2, and KMO enzymes and the kynurenine metabolite have been shown to be involved in the pathogenesis of neuropathic pain and other painful conditions (migraine, cluster headache, etc.) as well as depressive behavior. We highlighted the analgesic potential of novel agents targeting the enzymes of the kynurenine signaling pathway to explore their efficacy in both future basic science and transitional studies. Upcoming studies conducted on animal models will need to take into consideration the differences in TRP metabolism between human and non-human species. Since chronic painful conditions and depression have common pathophysiological patterns, and the kynurenine signaling pathway is involved in both of them, future clinical studies should aim to have outcomes targeting not only pain, but also functionality, mood changes, and quality of life.

## 1. Introduction

L-tryptophan (i.e., Trp) is an aromatic amino acid that was first discovered in 1901 when it was extracted from a hydrolyzed casein [1]. Trp is produced by bacteria and fungi, and requires obligatory dietary intake in humans (essential amino acid). This amino acid is required for protein synthesis and tryptamine production, but it also undergoes hydroxylation for the production of 5-hydroxytryptamine (5-HT, serotonin) in the brain and the gut via the enzyme tryptophan hydroxylase (TPH) [2]. The neurotransmitter (NT) serotonin is implicated in endogenous nociceptive information processing, both in a descending inhibitory and descending facilitatory fashion within the spinal cord. There is evidence that supports the analgesic properties of serotonin-modulating antidepressants (e.g., selective-serotonin reuptake inhibitors) in a number of chronic pain disorders [3]. However, the knowledge behind the exact mechanism of action of serotonin in chronic pain in humans is still insufficient. Furthermore, serotonin serves as an intermediate metabolite in the production of melatonin, a reaction occurring in the pineal gland catalyzed by the *N*-acetyltransferase (NAT) and *N*-acetylserotonin *O*-methyltransferase (ASMT) enzymes [4]. Finally, amino acid TRP can undergo a wide array of catabolic reactions collectively known as the kynurenine pathway. At this point, TRP and its downstream metabolites assume, among others, an important role in energy production, immune system regulation, pain perception, and the pathogenesis of many psychiatric conditions [5]. The purpose of this review is to discuss the role of TRP and the kynurenine signaling pathway in different chronic pain conditions and frequently associated depression.

## 2. The Catabolic Pathway of Tryptophan

The kynurenine pathway is a complex metabolic process governed primarily by the enzymes indoleamine 2,3-dioxygenase (IDO) and, to a lesser extent, by tryptophan 2,3-dioxygenase (TDO), resulting in the formation of *N*-formyl-l-kynurenine (*N*-formyl-l-KYN) [6,7,8]. The expression of the indoleamine 2,3-dioxygenase 1 (IDO-1) enzyme takes place in peripheral tissues (macrophages, dendritic cells (DCs)) and the central nervous system (CNS) (microglia) immune cells, and is stimulated by an underlying infection and associated pro-inflammatory cytokines, such as interferon-γ (IFN-γ), tumor necrosis factor α (TNF-α), and interleukins (IL) 6 (IL-6) and 1β (IL-1β) [6]. The IFN-dependent mechanism of IDO-1 activation includes the binding of IFN-γ to a specific surface receptor, thus activating janus kinase 1 and 2 (JAK1/2), which further phosphorylates signal transducer and activator of transcription 1 (STAT1) at the tyrosine 701, although a similar process also takes place at serine 727 using protein kinase C-δ (PKCδ) [9]. The resultant STAT1-STAT1 homodimers translocate into the nucleus, binding to one of two IFN-γ-activated sites (GAS). This is synchronous with the binding of IFN-γ-regulated factor-1 (IRF-1) to one or both of the IFN-sensitive response elements (ISREs) [6]. The second, IFN-independent, mechanism of IDO-1 enzyme activation stems from an interaction between B7 complex found on DCs and cytotoxic T-lymphocyte antigen-4 (CTLA-4) on regulatory T-cells (Treg), and is maintained via transforming growth factor-β (TGF-β) and the non-canonical nuclear factor-κB (NFκB) signaling pathway [10]. More than a decade ago, Metz et al. demonstrated the existence of the indoleamine 2,3-dioxygenase 2 (IDO-2) enzyme, and found its tissue pattern expression similar to that of IDO-1 [11]. TDO represents another important enzyme of the kynurenic pathway that is expressed in the liver and which is regulated through stress-induced activation of the hypothalamic pituitary adrenal (HPA) axis [12,13], which is considered a protective mechanism to prevent the accumulation of indole, a toxic metabolite of TRP [14]. The first catabolic substrate of TRP metabolism, *N*-formyl-l-kynurenine (*N*-formyl-l-KYN), is metabolized into kynurenine (KYN) via kynurenine formamidase. KYN exhibits immunoregulatory effects by activating the aryl hydrocarbon receptor (AhR) found in DCs and T-lymphocytes [15]; however, it may also be a substrate for the enzymes kynurenine 3-monooxygenase (KMO), kynurenine aminotransferases (KATs) I-IV isoforms, and kynureninase (KYNU). In mammalian brains, KATs catalyze an irreversible transamination of KYN to produce kynurenic acid (KYNA), an *N*-methyl-d-aspartate (NMDA) antagonist [16]. A recent review provided no solid, reliable evidence that KYNA acts as an antagonist on acetylcholine nicotinic receptors [17]. Additionally, KYNA has the dual potential to facilitate (nanomolar to micromolar concentrations) α-amino-3-hydroxy-5-methyl-4-isoxazolepropionic acid (AMPA) receptors, and antagonize (millimolar concentrations) glutamate receptors [18]. Indeed, Rózsa et al. confirmed this by measuring field excitatory postsynaptic potentials (fEPSPs) in hippocampi of rat models during and after KYNA administration. [19] At different concentrations, KYNA caused slight (1 μM) and very slight (500–1000 nM) decreases in fEPSP amplitudes, which was in contrast to their increase (<500 nM) and maximum facilitation (250 nM) [19]. On the other hand, KYN is an appropriate substrate for the KYNU enzyme to catalyze the synthesis of anthranilic acid (ANA) [20]. Using KYN as a substrate, KMO catalyzes the production of 3-hydroxykynurenine (3-HK), which is then metabolized by enzyme kynureninase to derive 3-hydroxyanthranilic acid (3-HA). These two metabolites have been associated with both anti- and pro-oxidant properties [21]. The condensation of two 3-HA molecules results in the formation of cinnabarinic acid, a partial agonist of a type-4 metabotropic glutamate (Glu) receptor (mGlu4) [22]. 3-HK can be converted to xanthurenic acid (XA), an mGlu2/3 receptor activator [23], via the enzyme kynurenine aminotransferase. An enzyme 3-hydroxyanthranilate dioxygenase (HAOO) converts hydroxyanthranilate to 2-amino-3-carboxymuconate semialdehyde (ACMS), a substrate appropriate for spontaneous, non-enzymatic closure of the pyridine ring to form quinolinic acid (QA), or for the synthesis of 2-aminomuconate semialdehyde (AMS) via the enzyme ACMS decarboxylase (ACMSD) [24]. QA has demonstrated a strong neuronal toxicity potential through its selective NMDA receptor (NMDAR) agonist properties, pro-oxidant characteristics, ability to increase the concentration of glutamate in the neuronal microenvironment, potentiation of the effects of other excitotoxins, and its ability to change the structure of the blood–brain barrier [25]. Under the enzymatic activity of quinolinate phosphoribosyltransferase, QA undergoes a catabolic process to produce NAD(P)^+^ [26]. In contrast, AMS may enter the glutarate metabolic pathway for complete oxidation, or be nonenzymatically converted to picolinate.

**Figure 1:**
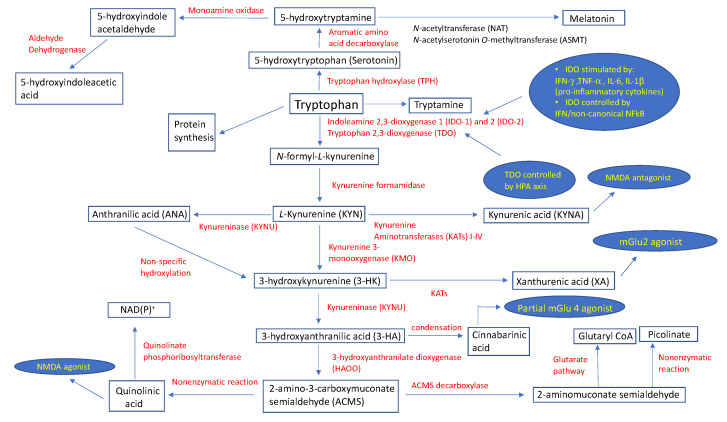
Schematic description of tryptophan metabolic pathways with assigned physiological roles and regulatory points of select metabolites. Abbreviations: HPA, hypothalamic pituitary adrenal; IFN-γ, interferon-γ; IL-1β, interleukin IL-1β; IL-6, interleukin IL-6; mGlu2, metabotropic glutamate receptor 2; mGlu4, metabotropic glutamate receptor 4; NAD(P)^+^, nicotinamide adenine dinucleotide phosphate; NFκB, nuclear factor-κB; NMDA, *N*-methyl-d-aspartate; TNF-α; tumor necrosis factor α.

The catabolic pathway of TRP with associated enzymes and their biologic functions is shown in Figure 1.

## 3. Variability of Kynurenine Metabolism between Human and Non-Human Species

A very important aspect about the kynurenine pathway is the variability of this process between different species and cell types. A review by Murakami and Saito explored these variations under both physiologic and inflammation-induced conditions [27]. The authors discussed the results of a previously conducted study that measured the activity of key kynurenine pathway enzymes in different tissues of rats, mice, gerbils, and rabbits [28]. Under physiological conditions, the activities of lung IDO-1 (rabbits) and brain KMO (gerbils) enzymes were 146–516 and 12.3–23.2 times higher, respectively, than those of other species. However, a lipopolysaccharide (LPS)-induced increase in IDO-1 activity was highest in the lungs of gerbils (30-fold) vs. mice (18-fold) vs. rats (several fold higher). The administration of LPS also significantly increased the concentration of QA in many tissues (e.g., brain, lungs, etc.) in gerbils; however, this was not seen in rat models, which is consistent with their minimal expression of IDO-1 in these tissues [27,29,30]. Similar findings between these two species were also seen in hippocampi following bilateral carotid artery occlusion, which prompted an inference that rat models do not replicate the responses to immune activation seen in humans. In summary, gerbils and humans share similar immune activation patterns following ischemic brain injury or LPS administration, as evidenced by increased QUIN levels in brain and systemic tissues.

In vitro studies have shown that not all human cells equally respond to stimuli or produce substrates of the kynurenine enzyme pathway [27,31]. For example, IFN-γ stimulated the IDO-1 expression and L-KYN production in blood macrophages, monocytes (THP-1), astrocytes, liver (SK-HEP1) and lung cells. However, the same substrate was produced only by a limited number of cells when LPS (blood macrophages and THP-1) and TNFα (blood macrophages) were applied. Moreover, in response to IFN-γ, LPS, and TNFα, only blood macrophages, THP-1 and SK-HEP1 cells were able to synthesize QUIN. LPS stimulated gerbil, but not rat brain microglia, astrocytes, and peripheral monocytes to produce QUIN [27,30]. Baseline KMO activity was recorded in these three cell types, but when IFN-γ was applied, small, but significant KMO upregulation occurred only in SK-HEP1 cells.

One possible explanation behind the variations in TRP metabolism could be the difference in nitric oxide (NO) synthesis between human and non-human species. NO is a small signaling molecule produced by nitric oxide synthase (NOS), which utilizes certain substrates and cofactors [nicotinamide-adenine-dinucleotide (NAD), flavin adenine dinucleotide (FAD), flavin mononucleotide (FMN) and tetrahydrobiopterin (BH_4_)] [32]. Human monocytes and macrophages have a very limited capacity to synthesize BH_4_, and instead rely on the production of a large amount of neopterin [33]. In contrast, macrophages in mice produce BH_4_ and use arginase to synthesize NO [34]. These variations in NO production apply not only to human and mice macrophages, but to other species as well [34]. NO regulates the IDO enzyme in three distinct aspects: by preventing IFN-γ-induced expression, by directly inhibiting the IDO, and by degrading the IDO through the proteasome pathway [35]. Noteworthy, the expression of key regulatory enzymes of kynurenine (IDO-1) and tetrahydrobiopterin (GCH1) pathways is regulated by common pro-inflammatory mediators, such as IFN-γ and LPS [36]. Pickert et al. analyzed the analgesic properties and morphine-related adverse effects of diaminohydroxypyrimidine (DAHP), an inhibitor of guanosine triphosphate cyclohydrolase 1 (GCH1) necessary for BH_4_ synthesis, in a mice cancer pain model [37]. The authors observed decreased nociceptive hypersensitivity, without evidence of morphine-induced respiratory depression. Further analysis revealed that the GCH1-product, BH_4_, was found responsible for inducing hyperalgesia and potentiating respiratory depression with morphine use. Collectively, nitric oxide production and ultimately TRP metabolism exhibit substantial variations between human and non-human species.

## 4. Kynurenine Pathway in the Pathogenesis of Neuropathic Pain

As per the International Association for the Study of Pain (IASP), neuropathic pain is defined as “pain caused by a lesion or disease of the somatosensory nervous system [38]”. Depending upon what part of nervous system has been affected by trauma, neuropathic pain is broadly classified into central or peripheral. Individuals may complain of concomitant positive (allodynia, paresthesias) and/or negative (impaired perception to various stimuli) somatosensory symptoms. Spinal cord microglia, a non-neuronal cell population, are in close neuroanatomical and neurochemical proximity with spinal cord neurons in terms of initiating and maintaining the neuropathic pain-associated hypersensitivity [39,40,41]. The relationship between the activity of several kynurenine pathway enzymes (IDO1/2, TDO, KMO, KYNU, and HAOO) and the development of neuropathic pain has been explored in a chronic constriction injury (CCI) rat model at the spinal cord and dorsal root ganglia (DRG) levels [42]. The authors measured the concentration of these enzymes in LPS-stimulated, microglial cells retrieved from cerebral cortices, and found that all the enzymes, aside from TDO, were derived from this cell population. Seven days after CCI, the mRNA expression levels of IDO-2, KMO, and, to a lesser extent, HAOO were shown to be upregulated at the spinal cord level, with no significant TDO mRNA level changes observed. The observed events occurred simultaneously with the activation of microglia/macrophages. Minocycline, an inhibitor of these cell lineages, was repeatedly injected intraperitoneally (i.p.) before and after the CCI, which decreased the levels of IDO-2 and KMO enzymes, parallel to diminished tactile and thermal hypersensitivity. Furthermore, IDO-2 and KMO enzyme inhibitors, 1-methyl-d-tryptophan (1-d-MT) and UPF 648, respectively, significantly attenuated hypersensitivity to mechanical and thermal stimuli. This provided strong evidence of the involvement of IDO-2 and KMO enzymes in the pathogenesis of neuropathic pain.

The modulatory effect of L-KYN was examined in rats with tactile allodynia previously subjected to L5–L6 spinal nerve root ligation [43]. In order to preserve intracerebrospinal fluid concentrations of L-KYN, this metabolite was administered in conjunction with probenecid, an organic anion transport inhibitor. Both intrathecal (i.t.) administration of L-KYN (1–30 μg), as well as i.p. injection of L-KYN (50–200 mg/kg) and probenecid (100 mg/kg) combination, significantly reversed tactile allodynia. The authors were in agreement that it was KYNA, an L-KYN metabolite, that mediated these effects by interacting with NMDARs. In a study using a neuropathic mice model, the expression of KMO, KYNU, and HAOO mRNAs was found to be increased in NeuN (neuronal nuclear antigen)-positive neurons in the contralateral hippocampal dentate gyrus [44]. Consistent with these elevated enzymes, also observed was the increased QA/KYN ratio. Spared nerve injury (SNI)-induced depressive-like behavior, but not mechanical allodynia, was attenuated upon intracerebroventricular (i.c.v.) administration of either KMO inhibitor, Ro 61-8048, or IL-1 receptor antagonist (IL-1RA); however, another study using rats showed that if the route of administration of Ro 61-8048 had been an i.t. injection, mechanical allodynia would have been alleviated [45]. This highlights the importance, in particular, of the IL-1 signaling pathway for these events, probably via the KMO-mediated pathway, which is in accordance with the increased IL-1β mRNA expression observed in the contralateral prefrontal cortex, but not in the hippocampus [44]. The IL-1β signaling pathway in the spinal cord has also been proposed as pivotal for the development of peripheral (liver) IDO-1-mediated, SNI-induced mechanical allodynia and depression-like behavior [46]. An experiment with i.p. administered LPS in gerbils, where the majority of available KYN measured in the CNS had been synthetized in the blood, may be able to explain the previous study’s findings that elevated IDO-1 levels were documented in the liver, and not in the CNS [47]. However, when cerebral inflammatory activity was induced using intrastriatal LPS delivery, nearly all measured KYN was produced locally in the brain.

## 5. Kynurenine Pathway and Association with Chronic Pain and Depression

Chronic pain and depression represent two notorious co-existing medical conditions, with data showing depression being present in up to 85% of patients suffering from various pain states [48]. The underlying pathophysiologic mechanisms behind this symbiosis involve shared aspects including anatomical structures, NTs, and signaling pathways. One study found a common neural pathway behind psychological pain, a precursor of depressive behavior, and physical pain, suggesting one theory of pain-induced depression [49]. In addition, the pathogenesis of depression is largely influenced by inflammatory cytokines, which provides an explanation as to why major depressive disorder (MDD) and inflammatory diseases often co-exist [50]. Recently, a review summarized the existing evidence regarding the role of the kynurenine pathway in the pathophysiology of neurocognitive and psychiatric diseases [51]. In particular, the inflammation hypothesis for Alzheimer’s disease (AD) discusses how an injury potent enough to recruit inflammatory brain cells then causes an imbalance between pro- and anti-inflammatory cytokines [51,52]. In this state of chronic inflammation, the concentration of several biomarkers including IL-1β, IL-1RA, IL-6, interleukin 10 (IL-10), and TNF-α is elevated in both plasma and CSF [51,53]. Hence, it is plausible that systematic inflammation may predispose, induce, or contribute to dementia. Relevant to the scope of the present review, patients with MDD had lower levels of neuromodulatory (TRP, KYN, and KYNA), alongside high neurotoxic (QA) kynurenine pathway levels [51,54,55,56]. The anti-inflammatory, antioxidant, anti-excitotoxic and immunomodulatory properties of KYNA make this endogenous metabolite pivotal in the pathogenesis of many mental disorders, including AD and MDD.

Huang et al. evaluated the relationship between serum TRP level and the quality of life of patients with colorectal adenocarcinoma with and without liver metastases [57]. The authors documented that significantly higher TRP concentration decline in metastatic vs. primary colorectal carcinoma. In order to better understand the burden of malignancy on the quality of life, the patients were asked to complete a series of questionnaires including the Rotterdam Symptom Checklist (RSC), Sickness Impact Profile (SIP), and the Hospital Anxiety and Depression (HAD) scale. Serum TRP was observed as an independent predictor of both RSC physical and SIP scores; however, no correlation was seen for RSC psychological or HAD anxiety/depression scores. Capuron et al. performed a study to try to establish a link between the quality of life and the serum levels of vitamin E, inflammatory markers (IL-6, C-reactive protein), and TRP in the elderly population [58]. Unsurprisingly, higher concentrations of inflammatory markers corresponded to lower TRP levels. However, higher TRP and vitamin E levels positively correlated with better physical and mental health status, the latter of which was especially pronounced.

Lawson et al. observed that i.c.v. administration of LPS produced depressive-like behavior mediated by central (brain), but not peripheral, IDO-1 upregulation and elevated KYN levels [59]. However, such aberrant behavior was avoided when an IDO-1 inhibitor, 1-d-MT, was administered in the same fashion in both IDO-1 knockout (KO) or wild-type (WT) mice. Pretreatment with minocycline or 1-d-MT in mice with systemic, LPS-induced inflammation reflected significant changes with respect to KYN concentrations [60]. Not only did the authors fail to observe depressive-like behavior in the treated mice group, but they also found a normalized KYN/TRP ratio in both plasma and the brain. Similarly, Deng et al. showed that pretreatment of a gentiopicroside (Gent 50 mg/kg, i.p.) once a day for three consecutive days prevented expression of depressive behavior through downregulation of LPS-induced GluN2B-containing NMDARs in the prefrontal cortex (PFC) [61]. At the same time, Gent prevented over-activation of the IDO enzyme and resulted in restored levels of IL-1β and TNF-α cytokines in the basolateral amygdala and PFC regions.

Research with preclinical models has deemed yet another proinflammatory cytokine, IL-6, relevant in the pathophysiology of both neuropathic pain and depression [62,63]. Using real-time PCR, the upregulation of IDO-1 mRNA was observed in the contralateral hippocampus in Wistar rats exhibiting right complete Freund’s adjuvant (CFA)-induced hind paw arthritis and depressive behavior simultaneously [64]. The molecular basis behind these observations probably lies in lower 5-HT and higher QA levels seen in IDO-1 upregulation. In contrast, resection of the tibial and common peroneal nerves (SNI) in mice, manifesting with mechanical hyperalgesia and depressive behavior, resulted in significant liver, but not hippocampal, IDO-1/IL-1β mRNAs expression [46]. The rationale as to why there were no detectable IDO-1 levels in the hippocampus could be explained by dramatically less systemic inflammation seen with SNI- vs. CFA-induced inflammation models. The SNI-induced inflammatory activity in the spinal cord has been attributed to increased IL-1β levels observed in the same location. Pain and depression, alongside liver expression of IDO-1/IL-1β mRNAs, were found to have subsided following an i.t. administration of an IL-1RA. However, only depressive behavior seemed to be alleviated in IDO-1-knockout mice, while symptoms of mechanical allodynia remained unaffected, implying that the occurrence of these comorbidities may possibly be IDO-1-independent. Collectively, this would indicate that the role of IDO-1, with respect to pain in a low-grade inflammatory state, was negligible. In CFA-treated mice, both nociception and depressive behavior were significantly attenuated upon administering 1-d-MT in the contralateral hippocampus, suggesting this as being the crucial location with respect to IDO-1 activity [64]. However, when acetaminophen was administered i.p., the authors noted a significant reduction in the nociception sensation (mechanical allodynia and thermal hyperalgesia), but not depressive behavior or IDO-1 mRNA expression, in arthritic rats vs. control. Under similar experimental conditions as for Wistar rats, the authors tested IDO-1 knockout mice and proved that they exhibited reductions in both nociceptive and depressive symptoms; however, both IDO-1 KO and WT mice retained high hippocampal IL-6 mRNA levels, suggesting synthesis upstream of IDO-1 regulation [64].

In addition to the aforementioned endogenous activators of the IDO enzyme, research using mice has also attributed acute influenza A virus (IAV) and chronic murine leukemia retrovirus (MuLV) with similar properties [65]. Previously, one study found that IAV was able to induce the activity of the IDO enzyme in the lungs and associated draining (mediastinal) lymph nodes [66]. In the referenced study, on the first day of respiratory infection with IAV, acute (transient) pain hypersensitivity as measured by reduced paw withdrawal threshold was observed, and persisted until one-week post-infection. However, this was not seen in IDO-1 knockout mice, suggesting the need for IDO-1 genes to be expressed for the observed phenomenon. The same study validated this genetic assumption in a single subset of MuLV-infected, IDO-1-producing spleen cells, termed CD19^+^ DCs. Similar to IAV, MuLV also produced a growing pain sensitivity until 18 days post-infection, but with high levels sustained afterwards. No IDO enzyme activity was seen in MuLV-infected, IDO-1 knockout mice. Still, both wild type and IDO-1 knockout mice exposed to MuLV had experienced increased pain sensitivity, although with a magnitude of observed effects higher in the wild-type control. Moreover, it was found that IDO-1-deficient, healthy mice receiving spleen cells from IDO-1-expressing, MuLV-infected mice also exhibited pain hypersensitivity. Exposing mice specimens to a TRP metabolite, KYN, resulted in greater increases in pain sensitivity among MuLV-positive vs. naive mice, suggesting a possible synergistic effect of KYN with the existing infection-induced cytokines.

Kynurenine 3-monoxygenase (KMO) has been proposed as another enzyme of interest in the TRP metabolic pathway relevant for the co-occurrence of depression and pain [44]. A study found that LPS-induced depressive-like behavior in mice was mediated by activating NMDARs owing to increased levels of QA [67]. A later study showed that LPS-treated mice with genetic deletions of KMO and HAOO enzymes exhibited no such behavior [68]. Similar principles behind QA-NMDAR interaction with respect to the co-occurrence of pain and depression were also amenable to another NMDAR antagonist, ketamine, which alleviates both neuropathic pain (NP) and NP-induced depression [69,70]. This dual action of ketamine is possible because of the relevant receptors for the previously mentioned conditions being located in distinct anatomical regions [44,71,72]. In another study, both chronic pain and concomitant depressive-like behavior in mice were significantly attenuated three weeks following oral administration of trans-astaxanthin, a naturally occurring molecule [73]. These changes coincided with a reduction in pro-inflammatory cytokines in the spinal cord, as well as increased 5-HT/5-HIAA and decreased KYN/TRP ratios in both the spinal cord and hippocampus. However, an i.p. injection of P-chlorophenylalanine over the course of five days terminated the observed anti-nociceptive and anti-depressive effects. Of note, the authors recorded higher thresholds for thermal hyperalgesia/mechanical allodynia and depressive-like behaviors consistently for days after cessation of trans-astaxanthin administration.

**Figure 2:**
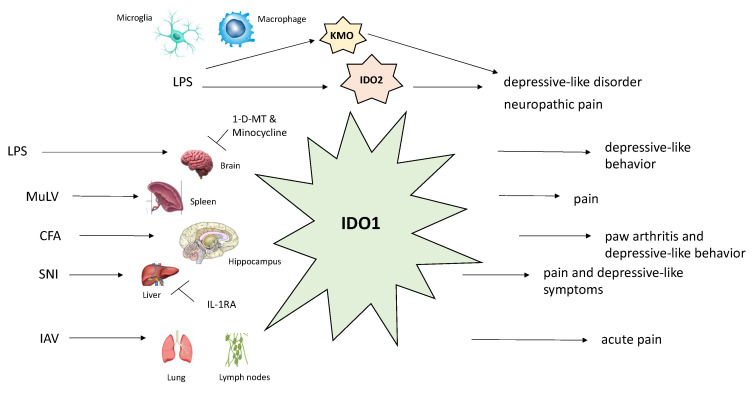
Schematic description of the effects of different stimuli on biological functions of major kynurenine pathway enzymes (IDO-1, IDO-2, KMO). Abbreviations: CFA, Complete Freund’s Adjuvant; IAV, influenza A virus; IDO-1, indoleamine 2,3-dioxygenase 1; IDO-2, indoleamine 2,3-dioxygenase 2; KMO, kynurenine 3-monooxygenase; LPS, lipopolysaccharide; MuLV, murine leukemia retrovirus; SNI, spared nerve injury.

A schematic representation of the major enzymes of the kynurenine pathway and their relationship with biological functions is shown in Figure 2.

## 6. Kynurenine Pathway and Headaches

Cluster headaches (CH) are characterized as unilateral headache attacks occurring several times a day and are accompanied by ipsilateral autonomic dysregulations (rhinorrhea, lacrimation, conjunctival injection, etc.). Not only are these attacks in close proximity to the circadian rhythm regulation, occurring frequently at night, but they are linked to seasonal changes as well. Three distinct anatomical structures have been proposed as being relevant for these attacks to occur: the parasympathetic nerve fibers, the trigeminovascular system, and the hypothalamus [74]. On the other hand, migraines represent a multifactorial, neurovascular brain disorder manifesting as recurrent attacks of debilitating headaches. Two major subtypes of migraines have been described: headache attacks adjunct to nausea, vomiting, and hypersensitivity to light and sound (migraine without aura), or with transient neurological symptoms (migraine with aura). However, the evidence from genetic studies and pharmacotherapeutic efficacy in both migraine subtypes suggests that these may represent a single condition with a variable pattern of clinical expression [75,76,77,78,79,80,81]. A central role behind the neurophysiology of a migraine attack stems from the activation of the trigeminovascular system, a complex roughly comprised of the trigeminal cranial nerve (CN V), associated nuclei, and cerebral blood vessels [82]. The nociceptive axonal projections (C-fibers and Aδ fibers) contained within the CN V release several important neuropeptides, such as calcitonin gene-related peptide (CGRP), neurokinin A, substance P, and pituitary adenylate cyclase-activating peptide (PACAP) [82,83,84,85]. Of these, CGRP has been found to facilitate the nociceptive transmission responsible for the perceived painful sensation of headache [86]. In addition, one of two PACAP forms (PACAP_1-38_; a 38-amino-acid neuropeptide), along with its receptors, may be involved in the pathogenesis of migraine and cluster headache, as evidenced by elevated PACAP-38 plasma levels during ictal phases of both painful conditions [87,88,89,90]. A randomized, double-blind study showed that intravenous (i.v.) administration of PACAP_1-38_ in healthy subjects and patients with migraine without aura produced headache and vasodilation in both groups, with the addition of delayed migraine-like attacks in the migraine group. Kortesi et al. [90] performed an in vitro study investigating the link between the kynurenine system and PACAP expression, focusing in particular on KYNA analog as a potential therapeutic agent in migraine. Rat models [91] were pretreated with i.v. NMDAR inhibitors [KYN, KYNA-a (KYNA synthetic analog), or MK-801] or saline prior to electrical stimulation of the trigeminal ganglion. As a result of the stimulation, significant mRNA expression of preproPACAP, PACAP_1-38_, and PACAP_1-38_-LI (mono-I labeled PACAP_1-38_) was documented in the caudal trigeminal nucleus (TNC); however, these increases were prevented using all pretreatment options other than saline. Thus, the authors provided the evidence that by modulating the glutamatergic transmission using known inhibitors or experimental KYNA derivatives, the overexpression of PACAP, as a valid biomarker of migraine, would be prevented. Greco et al. [91] provided evidence of the potential of KYNA-analogue 1 (KYNA-A1) in the management of migraine [92]. Using a preclinical rat model, pretreatment with tNTG followed by the plantar and orofacial formalin test, the authors observed an increased mRNA expression of nitric oxide synthase (nNOS), CGRP, and pro-inflammatory cytokines in the trigeminal ganglia and TNC. However, these changes were prevented with KYNA-A1 application, strongly implying a glutamate-mediated migraine modulation at the central and peripheral levels. A study showed that administering CFA into rat dura mater produced the markers of noxious stimulation, phosphorylated extracellular signal-regulated kinases 1 and 2 (pERK1/2), and IL-1β, in the trigeminal ganglion [93,94,95]. A follow-up study showed that pretreatment with an i.p. injection of a novel KYNA analogue, named SZR72, was able to diminish the immunoreactivity of pERK1/2 and IL-1β [96]. Nagy-Grocz et al. used an i.p. injection of nitroglycerin (tNTG) to produce a neurogenic inflammation and downregulate some of the key enzymes of KP (TDO-2, IDO-1, KMO, and KYNU) in the TNC of rats [97]. The hyperactive NMDAR population was rendered a crucial finding in regard to this animal model’s migraine pathogenesis.

Various metabolites of the kynurenine pathway have been implicated in the pathophysiology of both CH and chronic migraines [98,99]. Observed in both of these painful conditions was the significant reduction in certain Trp downstream metabolites, including KYN, KYNA, 3-HK, 3-HANA, 5-HIAA, and QA. Indeed, these results confirm that unopposed excitation of NMDA receptors plays an important role in mediating pain in these conditions. An interesting finding in these studies was the documented increase in XA and ANA seen only in migraine patients; the authors suggested that elevated XA levels represent compensatory reinforcement of endogenous analgesic mechanisms through the activation of mGlu2 receptors, while the biologic significance behind compensatory high ANA levels remains unknown. The excitatory NT Glu has been deemed pivotal in deciphering migraine pathophysiology as well [100], with higher blood levels documented in both ictal and interictal periods [101]. Increased brain glutaminergic activity predisposes to greater susceptibility to cortical spreading depression (CSD), a wave of depolarization of neurons and glial cells [102]. In accordance with the long-standing belief that CSD has exclusively been related to migraine with aura, one would expect to find elevated glutamate levels in such a patient population; however, new evidence begs to differ [103]. A clinical trial evaluated the possibilities of modulating the aura phenomenon by comparing the Aurastop (a formulation of tanacetum parthenium, 5-hydroxytryptophan, and magnesium) vs. magnesium alone in 50 patients with migraine with aura [104]. The results were consistent with a greater number of patients exhibiting a >50% reduction in duration of aura (96% vs. 14%, *p* < 0.001) and a >50% reduction in aura-related pain disability (96% vs. 10%, *p* < 0.001) in patients taking Aurastop vs. magnesium, respectively. Increased metabolism of L-TRP following Aurastop intake and subsequently elevated levels of KYNA were likely responsible for the observed findings. Additionally, patients taking Aurastop had to take analgesics less frequently, but with greater benefit reported, than those taking magnesium. Immunohistochemistry analysis revealed an increased glutamate staining pattern in TNC of rats following a dural CFA administration; however, pretreatment with SZR72 correlated with a glutamate concentration observed in healthy rats [105].

Upstream of the kynurenine metabolic pathway, TRP metabolizes into serotonin, a NT implicated both in the pathogenesis [106,107] as well as therapy [108] in migraineurs. Cseh et al. measured elevated levels of not only glutamate, but also of KYN and KYNA in TNC following a CFA whisker pad injection [109]. Within the somatosensory cortex, significant increases in 5-HT and KYNA concentrations were found. KYN, KYNA, as well as 5-HT may represent a feedback mechanism to diminish glutamate sensitization and trigeminovascular pathway activation, respectively [110]. Of note, the observed metabolite concentration changes occurred in a narrow time interval between 24 and 48 h, which corresponds to central and peripheral sensitization of the trigeminovascular system and which paves a pathway for future antibody-based therapies [109,111,112,113,114].

## 7. Kynurenine Pathway and Other Conditions

An exploratory study investigated the concentrations of KYN and TRP in females suffering from the temporomandibular disorders myalgia (TMDM) and fibromyalgia [115]. In TMDM patients, the authors observed a positive correlation between the KYN/TRP ratio and the average and worst pain intensity, as well as a negative correlation between TRP plasma levels and the worst pain intensity. Moreover, patients with fibromyalgia had statistically significant lower concentrations of plasma TRP compared to controls, alongside the negative correlation observed between KYN/TRP ratio and anxiety levels [115].

## 8. Conclusions and Future Directions

In this review, we evaluated the role of the amino acid TRP and its metabolites in the pathogenesis of different chronic pain conditions. We also reviewed which substrates and enzymes within the TRP catabolic (kynurenine) pathway could serve as potential therapeutic targets. The summarized evidence shows the upregulation of IDO-1, IDO-2, KMO, KYNU, etc., in liver and CNS tissues in different neuropathic pain models. Preventing the cells from expressing (i.p. minocycline) or directly inhibiting (i.t. 1-d-MT/UPF 648) IDO-2/KMO enzymes resulted in decreased mechanical, tactile, or thermal hypersensitivity. We described the important link between IDO-1 upregulation, the IL-1β signaling pathway, serotonergic (hypoactive) and glutamatergic (hyperactive) transmission, and depression. Accordingly, we highlighted some new promising agents (trans-astaxanthin, gentiopicroside, Ro 61-8048, IL-1RA, etc.) and their possibility to be used as potential therapeutic drugs. We also observed inconsistent concentrations of different KYN pathway metabolites and discussed the clinical importance of PACAP_1-38_ in migraine and cluster headaches. Finally, we discussed the management of these two painful conditions by using agents modulating the activity of NTs glutamate (KYNA, KYNA analogues, and MK-801) and serotonin (Aurastop). A summary of pertinent results focusing on the role of the kynurenine pathway as well as prospective therapeutic agents in various clinical conditions are shown in Table 1.

The roles of TRP and its metabolites show a promising therapeutic potential in chronic pain conditions (neuropathic pain, migraines, and cluster headaches). Preliminary results have been encouraging to consider the possibility of developing new drugs with selective mechanisms of action and limited side effect profiles. Additional research is warranted to explore the potential of the kynurenine signaling pathway in both future basic science and transitional medicine studies. Upcoming studies focusing on the use of the kynurenine pathway in pain management conducted on animal models will need to take into consideration the differences in TRP metabolism between human and non-human species. Since chronic painful conditions and depression have common pathophysiological patterns, and the kynurenine signaling pathway is involved in both of them, future clinical studies should aim to have outcomes targeting not only pain, but also functionality, mood changes, and quality of life.

## Figures and Tables

**Table 1:** Summary of changes in the kynurenine metabolic pathway in different clinical conditions with possible therapeutic agents.

Clinical Condition	The Role of the Kynurenic Pathway
Neuropathic pain	Activation of microglia/macrophages with simultaneous upregulation of IDO-2/KMO at spinal cord level; Increased expression of KMO, KYNU, and HAOO in NeuN-positive neurons in contralateral hippocampal dentate gyrus; Increased liver IDO-1/IL-1β mRNAs expression; increased spinal cord IL-1β levels.
	Possible therapeutic agents: I.t. 1-d-MT (IDO-2 inhibitor) and UPF 648 (KMO inhibitor) administration ↓ mechanical and thermal hypersensitivity; I.p. minocycline injection ↓ IDO-2/KMO and ↓ tactile and thermal hypersensitivity; IL-1RA i.t. administration attenuated both pain and depression through ↓ liver IDO-1/IL-1β mRNAs expression; I.t. (L-KYN) and i.p. (L-KYN and probenecid) injection reversed tactile allodynia; this was mediated by KYNA on NMDARs.
Depression	CFA induces both depression and arthritis through IDO-1 upregulation (↓ serotonin, ↑ QA); LPS-induced behavior mediated by activating NMDARs from increased QA levels; SNI-induced mechanical allodynia and depression are mediated by IL-1β signaling pathway in the spinal cord and IDO-1 in the liver.
	Possible therapeutic agents: Oral trans-astaxanthin ↓ pain and depression by ↓ pro-inflammatory cytokines in the spinal cord and ↑ 5-HT/5-HIAA and ↓ KYN/TRP ratios in the spinal cord and hippocampus. I.p. P-chlorophenylalanine administration terminated these changes; Ro 61-8048 (KMO inhibitor) or IL-1RA i.c.v. administration attenuated depression in the SNI-induced model; LPS administration (i.c.v.) induced depression mediated by increased IDO-1 and KYN levels in the CN; however, 1-d-MT (IDO-1 inhibitor) administered in the same fashion prevented depression; I.p. gentiopicroside prevented depression and IDO-1 overactivation by downregulating LPS-induced GluN2B-containing NMDARs in the prefrontal cortex; 1-d-MT decreased both pain and depression in the CFA model.
Headache	↑ XA and ANA in migraine patients; XA represents endogenous analgesic metabolite acting upon mGlu2 receptors; CFA whisker pad injection led to ↑ KYN, KYNA, and glutamate in TNC as well as ↑ 5-HT and KYNA in the somatosensory cortex; I.p. injection of NTG produces a neurogenic inflammation by downregulating IDO-1, TDO-2, KMO, and KYNU enzymes in TNC; Reduction in levels of KYN, KYNA, 3-HK, 3-HA, 5-HIAA, and QA in migraine and cluster headaches; 5-HT, KYN, and KYNA may serve to diminish glutamate sensitization and trigeminovascular activation; PACAP_1-38_ levels are elevated during ictal phases of migraine and CH; i.v. PACAP_1-38_ induced headache in both migraineurs (without aura) and the control group, as well as delaying migraine-like headache in migraineurs.
	Possible therapeutic agents: KYNA-A1 i.p. application prevents expression of nNOS, CGRP, and proinflammatory cytokines in TNC and trigeminal ganglia in migraine; Aurastop (tanacetum parthenium, 5-HT, and magnesium) resulted in significant reduction in aura and aura-related pain disability consistent with ↑ KYNA; I.v. pretreatment with KYN, KYNA-a, and MK-801 decreased TNC PACAP_1-38_ levels by modulation of glutamatergic transmission.
Other conditions	Temporomandibular disorders myalgia: positive correlation between KYN/TRP and average/worst pain intensity; negative correlation between TRP and worst pain; Fibromyalgia: ↓ TRP; negative correlation between KYN/TRP and anxiety.

Abbreviations: IDO, indoleamine 2,3-dioxygenase; KMO, kynurenine 3-monooxygenase; KYNU, kynureninase; HAOO, 3-hydroxyanthranilate dioxygenase; NeuN, neuronal nuclear antigen; IL-1β, interleukin 1 beta; IL-1RA, interleukin 1 receptor antagonist; 1-d-MT, 1-methyl-d-tryptophan; CFA, complete Freund’s adjuvant; QA, quinolinic acid; LPS, lipopolysaccharide; i.c.v., intracerebroventricular; GluN2B, glutamate N2B; NMDAR, *N*-methyl-d-aspartate; XA, xanthurenic acid; ANA, anthranilic acid; mGlu2, metabotropic glutamate receptor 2; KYN, kynurenine; TNC, trigeminal nucleus caudalis; 5-HT, 5-hydroxytryptamine; NTG, nitroglycerin; TDO, tryptophan 2,3-dioxygenase; 3-HK, 3-hydroxykynurenine; 3-HA, 3-hydroxyanthranilic acid; 5-HIAA, 5-hydroxyindolacetic acid; nNOS, nitric oxide synthase; CGRP, calcitonin gene-related peptide; TRP, tryptophan; ↓, decrease; ↑, increase.
